# Interaction of Toll-Like Receptors with the Molecular Chaperone Gp96 Is Essential for Its Activation of Cytotoxic T Lymphocyte Response

**DOI:** 10.1371/journal.pone.0155202

**Published:** 2016-05-16

**Authors:** Weiwei Liu, Mi Chen, Xinghui Li, Bao Zhao, Junwei Hou, Huaguo Zheng, Lipeng Qiu, Zihai Li, Songdong Meng

**Affiliations:** 1 CAS Key Laboratory of Pathogenic Microbiology and Immunology, Institute of Microbiology, Chinese Academy of Sciences (CAS), Beijing, P.R. China; 2 Institute of Life Sciences, Jiangsu University, Zhenjiang, P.R. China; 3 Department of Microbiology and Immunology, Medical University of South Carolina, Charleston, SC, United States of America; University Paris Sud, FRANCE

## Abstract

The heat shock protein gp96 elicits specific T cell responses to its chaperoned peptides against cancer and infectious diseases in both rodent models and clinical trials. Although gp96-induced innate immunity, via a subset of Toll like receptors (TLRs), and adaptive immunity, through antigen presentation, are both believed to be important for priming potent T cell responses, direct evidence for the role of gp96-mediated TLR activation related to its functional T cell activation is lacking. Here, we report that gp96 containing mutations in its TLR-binding domain failed to activate macrophages, but peptide presentation was unaffected. Moreover, we found that peptide-specific T cell responses, as well as antitumor T cell immunity induced by gp96, are severely impaired when the TLR-binding domain is mutated. These data demonstrate the essential role of the gp96-TLR interaction in priming T cell immunity and provide further molecular basis for the coupling of gp96-mediated innate with adaptive immunity.

## Introduction

As a member of the heat shock protein 90 (HSP90) family, gp96 (glucose-regulated protein 94, GRP94) is one of the most abundant chaperones in the endoplasmic reticulum (ER). Both rodent models and clinical trials have demonstrated that gp96 purified from tumors or complexed with viral antigens in vitro elicits antitumor effects or antigen-specific humoral and CD8^+^ T cell (CTL) immunity against tumors and viruses [1–3]. The immunogenicity of gp96 is attributed to its ability to activate both the innate and adaptive immune responses.

First, together with HSP70 and HSP90 in the cytosol, gp96, TAP (transporter associated with antigen processing) molecules, and calreticulin in the ER are thought to constitute a relay line for antigenic peptide transfer from the cytosol to MHC class I molecules in a concerted and regulated manner [4, 5]. Under intradermal or subcutaneous immunization, the gp96-antigenic peptide complexes access the draining lymph node and are predominantly internalized by subsets of antigen presenting cells (APCs) through cell surface receptor CD91. Internalized gp96 can effectively present the associated peptides to MHC class I and class II molecules and thus activate specific CD8^+^ and CD4^+^ T cell responses [6, 7].

Second, gp96 itself binds to and acts as a master chaperone for Toll-like receptors (TLRs) (*e*.*g*., TLR2, TLR4, and TLR9) on APCs, stimulating pro-inflammatory and Th1-type cytokine (TNF-α, IL-1β, and IL-12) secretion [8, 9]. The TLR-2/4-binding domain of gp96 was recently mapped to its C-terminal loop structure [10]. In addition, gp96 also interacts with CD91, which leads to CD91 phosphorylation and activation of NF-κB and p38 MAPK. This allows for the maturation of APCs, releasing cytokines, and priming of T-helper (Th) cells [11]. Both of these events are believed to be important for gp96 to induce robust T cell responses. However, it is still unclear how the gp96-mediated innate immune response via TLRs functionally affects its CTL activation ability through antigen presentation.

Our previous studies have shown that gp96 complexed with antigens from tumors or hepatitis B virus (HBV) engages macrophages to cross-present antigens. This allows for activation of specific CTL responses and exhibits significant antitumor or antiviral effects [12–14]. Additionally, consistent with previous studies [8, 10, 15], we also found that gp96 binds to and activates the TLR2/4 pathway in regulatory T cells (Tregs) [16]. While sufficient data exist to demonstrate gp96-mediated innate and adaptive immune functions, direct evidence for the potential role of the gp96-TLRs interaction on T cell activation is lacking. Here, we further dissected the impact of the TLR activation activity of gp96 on its ability to induce T cell responses against cancer and viruses.

## Materials and Methods

### Ethics statement

Animal studies were carried out according to the guidelines set forth by the Institute of Microbiology, Chinese Academy of Sciences of Research Ethics Committee under the approved protocol numbers PZIMCAS2011001. All animal experiments were performed in strict accordance with institutional guidelines on the handling of laboratory animals. Mice were euthanized when the maximum tumor size (diameter: 2.0 cm) had been reached. Cervical dislocation was applied in the study to minimized animal suffering and distress. The health of the animals was monitored every other day and there was no unexpected deaths. To minimized mice suffering and distress, the only tumor was implanted in the subcutaneous site in the flank on each animal.

### Cell culture and antibodies

The CD8^+^ T cell hybridoma cell line B3Z, the melanoma cell line B16.F10, and H-2^b^ fibroblast cell line K41 were maintained in RPMI 1640 supplemented with 10% fetal calf serum (FCS) (GIBCO, NY, USA) and 100 U/ml streptomycin/penicillin. Raw264.7 cells and HEK293 cells (a cell line that does not express TLR2/TLR4) [17] were maintained in complete DMEM (GIBCO) supplemented with 10% fetal calf serum (FCS) and 100 U/ml streptomycin/penicillin. The cells were maintained at 37°C in an atmosphere containing 5% CO_2_.

The HBc_87–95_ (SYVNTNMGL), OVA8 (NH2-SIINFEKL-COOH), and OVA20 (NH2-SGLEQLESIINFEKLTEWTS-COOH) peptides were synthesized by GL Biochem Ltd. (Shanghai, China) to >95% purity. Soluble PE-HBc_87-95_ tetramers were synthesized by QuantoBio (Beijing, China). The following antibodies were obtained as indicated: anti-grp94, anti-His tag, anti-TLR2, anti-TLR4, anti-pIκB-α, anti-β-actin and anti-p65 antibodies were purchased from Santa Cruz Biotechnology (CA, USA). PerCP-Cy5.5-conjugated anti-mouse CD3, FITC-conjugated anti-mouse CD8, PE-conjugated anti-mouse CD4, and APC-conjugated anti-mouse IFN-γ antibodies were from eBioscience (San Diego, CA, USA), APC-conjugated anti-human CD11b antibody was from Biolegend (San Diego, CA, USA).

### Construction of recombinant gp96 vectors

The cDNA encoding the mature human gp96 in a pET28a ^(+)^ vector (Novagen, Madison, WI, USA) was used as a template for all PCRs. The following primers for gp96-mutant and gp96-deleted CBD (gp96-ΔCBD) were synthesized by Sangon as described previously [10], and the sequences of the primers were: sense, 5-TGGTGGCCAGCCAGTACGCAGCGTCTGCCGCCGCGGCGGCGATCATGAAAGCACAAGC-3; and antisense, 5-GCTTGTGCTTTCATGATCGCCGCCGCGGCGGCAGACGCTGCGTACTGGCTGGCCACCA-3’ (gp96-mut); sense, 5-GTGTGCTTTGGTGGCCAGCCAGTACATCATGAAAGCACAAGCGTACCAA-3; and antisense, 5-TTGGTACGCTTGTGCTTTCATGATGTACTGGCTGGCCACCAAAGCACAC-3 (gp96-ΔCBD). The 5’sense primer (5’ GGAATTCATG GGCAGCAGCCATCAT 3’) and the 3’sense primer (5’ GCTCTAGACTATTACAATTCATCTTTTTC 3’) were used to prepare regions flanked by 5’ *Eco*RI and 3’ *Xba*I restriction sites. All constructs were subcloned into the pFastBac1 vector. The Bac-to-Bac Baculovirus Expression System used to express the recombinant gp96 proteins was used as described previously without significant changes [18].

### Purification of recombinant protein

Soluble recombinant gp96 proteins were isolated as follows. The supernatant was collected and loaded onto a Ni-sepharose column (GE Healthcare, USA). After elution, the gp96 proteins were purified using a Hitrap Q HP column (GE Healthcare, USA). Additional purification was performed using Superdex 200 10/300 GL (GE Healthcare, USA). The purified gp96-wt, gp96-mut, and gp96-ΔCBD proteins were desalted and concentrated using an Amicon Ultra [15ml 50KD] (Millipore, USA) and stored at -80°C. Then, a portion of the gp96-wt, gp96-mut and gp96-ΔCBD preparations was labeled with FITC (Molecular Probes Inc, USA). The native gp96 (mgp96) was purified from healthy mouse livers, according to a protocol described previously [19].

### Isolation of primary human macrophage cells

Peripheral blood mononuclear cells (PBMCs) were isolated from fresh healthy blood by Ficoll-Paque PLUS. CD11b^+^ macrophage cells were purified from the PBMCs by FACS using BD FACS ARIA II SORP (BD Biosciences, USA).

### Western blot analysis

Cell lysates were collected in sodium dodecyl sulfate (SDS) sample buffer. Samples were separated by electrophoresis on 10% polyacrylamide gels and electrophoretically transferred to PVDF membranes (Millipore, USA). The membranes were probed with specific antibodies against TLR2, TLR4, phosphor-IκB-α (Ser32), His or β-actin for internal control. After a washing step, the membranes were incubated with secondary HRP-conjugated antibodies. The protein bands were visualized by enhanced chemiluminescence detection reagents (Applygen Technologies Inc., China).

### Immunofluorescence staining

Raw264.7 cells were stimulated for 30 min with 100 μg/ml gp96-wt, gp96-mut, or gp96-ΔCBD labeled with FITC. After permeabilization and blocking with 5% BSA for 30 min, cells were incubated with 5 μg/ml anti–NF-κB/p65 (Santa Cruz, USA), according to a protocol described previously [16]. Cell nuclei were stained with DAPI (Invitrogen, USA).

### Co-immunoprecipitation

Raw264.7 cells or human macrophage cells were incubated with PBS, 100 μg/ml of gp96-wt, gp96-mut, or gp96-ΔCBD at 4°C for 4 h. Equal amounts of anti-His Abs or control Ab were added to cell lysates and incubated overnight at 4°C. The supernatant was incubated with protein G beads for another 2 h, followed by extensive washing of the beads. Protein G beads were boiled in SDS-PAGE sample buffer to elute the immunoprecipitates for detection of TLR2 or TLR4 by Western blotting.

### Peptide binding assay

Binding of gp96 was quantified by ELISA assays. The solid-phase binding assay was performed as described previously with some modifications [20]. Briefly, 96-well streptavidin plates (Thermo Fisher Scientific, USA) were coated with the biotinylated peptide HBc_87–95_, and after blocking with 5% non-fat dry milk at 37°C, serial dilutions of mgp96, gp96-wt or gp96-mut protein, ranging from 0.03–200 μg/mL, were added to each well in 100 μL of binding buffer (20 mM HEPES (pH 7.2), 20 mM NaCl, 2 mM MgCl_2_, and 100 mM KCl) to allow for binding for 1.5 h. After, the plates were incubated with rat anti-grp94 antibody and HRP-conjugated goat anti-rat antibody. The substrate TMB (3, 3’, 5, 5’-tetramethylbenzidine) was used for detection. The reaction was measured at 450 nm as described [21].

### Antigen presentation assay with B3Z

Wild-type or mutant recombinant gp96 or native gp96-OVA20 peptide complexes were generated by incubating the mixtures of protein and peptide at 50°C for 10 min, followed by 30 min at room temperature. K41 cells (1×10^6^) were washed twice with warm RPMI 1640 and resuspended in 200 μL RPMI 1640 in a 24-well plate. The gp96-OVA20 peptide complexes (75 μM) were incubated with K41 cells at 37°C with 5% CO_2_ for 30 min. The cells were resuspended at a concentration of 1×10^6^/ml, and 100 μL cells were cultured with 100 μL 1×10^6^/ml B3Z cells over-night. The cells were washed once with 200 μL PBS and lysed by addition of 100 μL Z buffer (0.12 mM Chlorophenolred-beta-D-galactopyranoside (CPRG, Calbiochem, USA), 100 mM 2-mercaptoethanol, 9 mM MgCl_2_, and 0.125% NP-40 in PBS). After 4 h of incubation at 37°C, 50 μL stop buffer (300 mM glycine and 15 mM EDTA in water) was added, and the absorbance was read in an ELISA reader at 630 nm.

### Immunization of mice

Female C57BL/6 mice (6–8 weeks old) and female BALB/c mice (6–8 weeks old) were purchased from the Peking University Experimental Animal Center (Beijing, China). The various gp96–peptide complexes were prepared by incubating mgp96, gp96-wt and gp96-mut protein (20 μg) with HBc_87-95_ peptide or B16 Ag (50 μg each) first at 50°C for 10 min, followed by 30 min at room temperature. B16 Ag was prepared from B16-F10 cell lysates as described [12]. BALB/c mice were immunized by subcutaneous injection in the abdominal flank with mgp96, gp96-wt or gp96-mut-HBc_87-95_ peptide complexes (20 μg/mouse) or with the peptide alone as a control. After immunization at weeks 1, 2, and 4, the mice were sacrificed at week 5 for further analysis.

C57BL/6 mice (n = 15/group) were immunized with mgp96-, gp96-wt- or gp96-mut-B16 Ag complexes or B16 Ag alone as a control. After immunization at weeks 1, 2, and 4, the mice were subcutaneously inoculated with 5 ×10^4^ B16.F10 cells at week 5. Mice (n = 5/group) were sacrificed for ELISPOT assay in spleen on the 22^nd^ day after B16 injection, and the left mice (n = 10/group) were monitored till the 50^th^ day for the survival analysis. Tumor size was measured every other day with a Vernier caliper. Tumor volume was calculated using the formula: Tumor volume = length × width^2^/2.

### IFN-γ ELISPOT analysis

ELISPOT assays were performed using the BD ELISPOT Set from BD Biosciences. Briefly, isolated splenocytes (5×10^5^ cells/well) and B16 Ag or HBc_87-95_ peptide (10 μg/ml) were added to each well in triplicate and incubated at 37°C for 30 h. The spots were counted and analyzed with an ELISPOT Reader (Biosys, Germany).

### Flow cytometry and intracellular cytokine and tetramer staining

Intracellular IFN-γ staining assays were performed using a Cytofix/Cytoperm kit (BD Biosciences, USA). Splenocytes (2 ×10^6^ cells) were incubated with B16 Ag or HBc_87-95_ peptide (10 μg/ml) at 37°C for 4 h. After blocking, primary and secondary antibody staining was performed step by step. All the staining protocols were performed as described [14, 22, 23]. Tetramer staining and FACS analysis were performed as described [23]. Stained cells were analyzed using a FACS Calibur flow cytometer (BD Biosciences, USA) and the FlowJo Software (Tree Star Inc.).

### T cell cytotoxicity assay

*In vitro* CTL activity assays were performed as described [13]. The target cells (P815) pre-labeled with 5-(6)-carboxy-fluorescein succinimidyl ester (CFSE) were mixed with effector cells (mouse splenocytes) at various ratios in 96-well round-bottom plates. Then, they were incubated at 37°C for 5 h. Finally, samples were labeled with PI and analyzed by FACS using CellQuest software (BD Biosciences, USA).

### Statistical analysis

Statistical analyses of ELISPOT assays, cytotoxicity assays, relative mRNA expression levels, and tumor growth inhibition among Ag-, mgp96-, gp96-mut-, gp96-wt-immunized mice were performed by the two-tailed Student’s t test using GraphPad Prism 5.01. P values <0.05 were considered statistically significant.

## Results

### A client-binding domain gp96 mutant loses TLR activation capacity but retains peptide cross-presentation activity

**Fig 1:**
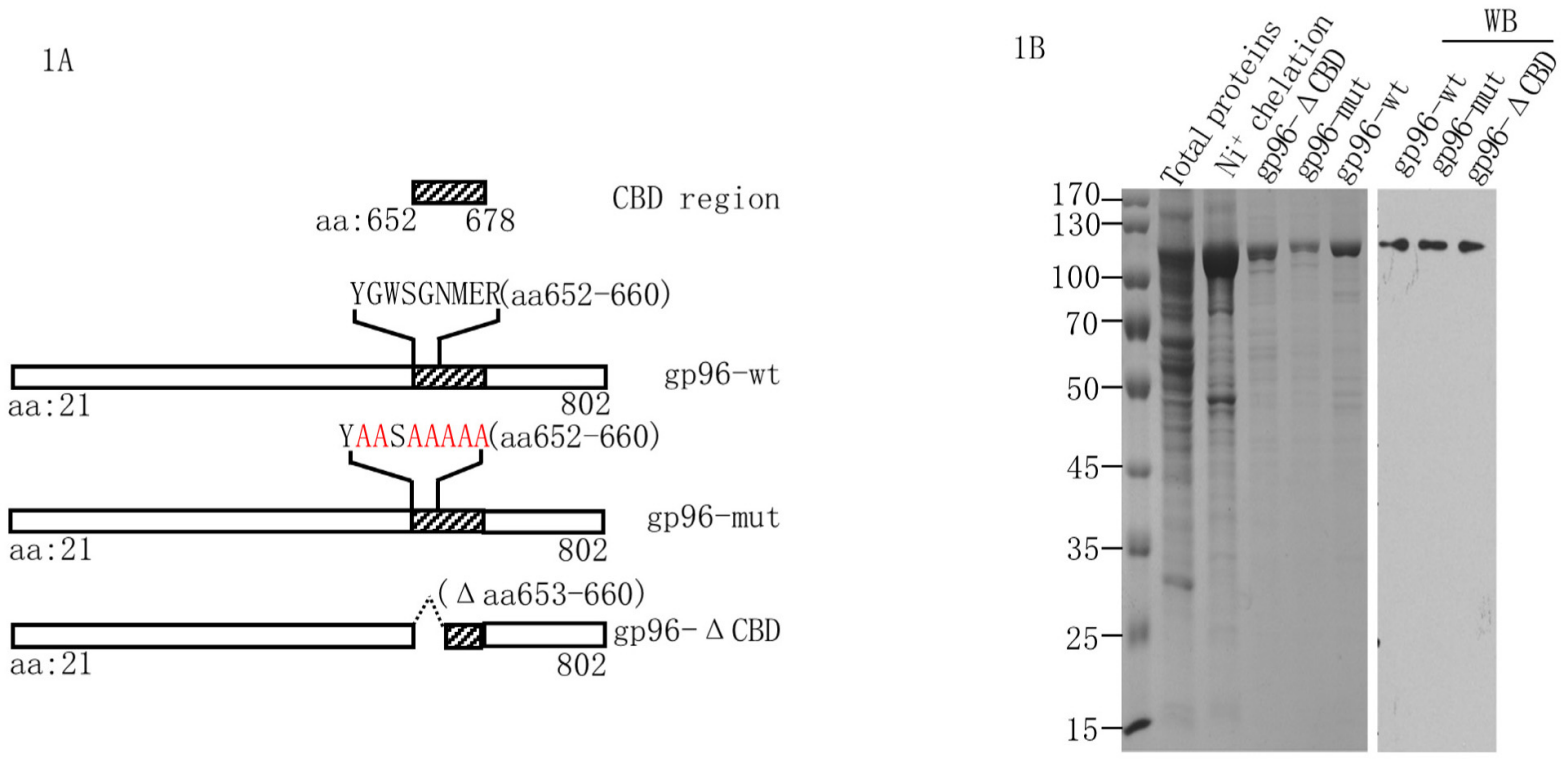
Construction and purification of recombinant gp96 and gp96 mutants. (A) Schematic representation of construction of recombinant gp96 proteins used in this study, wild-type gp96 (gp96-wt), mutated gp96 within the CBD region (gp96-mut) and gp96 mutant with deletion of aa 653–660 within the CBD region (gp96-ΔCBD). The CBD region box is shown, and residues in red are ones mutated into Ala. (B) Recombinant gp96-wt, gp96-mut and gp96-ΔCBD expressed in *Baculovirus* was purified by Ni+ chelation chromatography and anion-exchange chromatography using Hitrap Q column. The gp96 preparations were subjected to 10% SDS-PAGE and visualized with Coomassie staining and western blotting with an anti-gp96 antibody.

A previous study demonstrates that the GWXGNMER motif within the client-binding domain (CBD, aa 652–678) of gp96 is essential for its interaction with TLRs [10]. To study the function of gp96 in the activation of TLRs, we generated a deletion mutant of gp96 lacking its CBD region (gp96-ΔCBD) and introduced Ala substitution mutations at all of the conserved residues in the first half of the CBD (gp96-mut) as shown in Fig 1A. We have shown that eukaryotically expressed recombinant gp96 possesses similar peptide-binding parameters and T cell immune activity as that of native gp96 [21]. Thus, the wild type and mutant recombinant gp96 proteins were expressed using the baculovirus system. Preparations of purified gp96-wt, gp96-mut, and gp96-ΔCBD were detected by Coomassie staining and western blotting with an anti-Grp94 antibody (Fig 1B).

**Fig 2:**
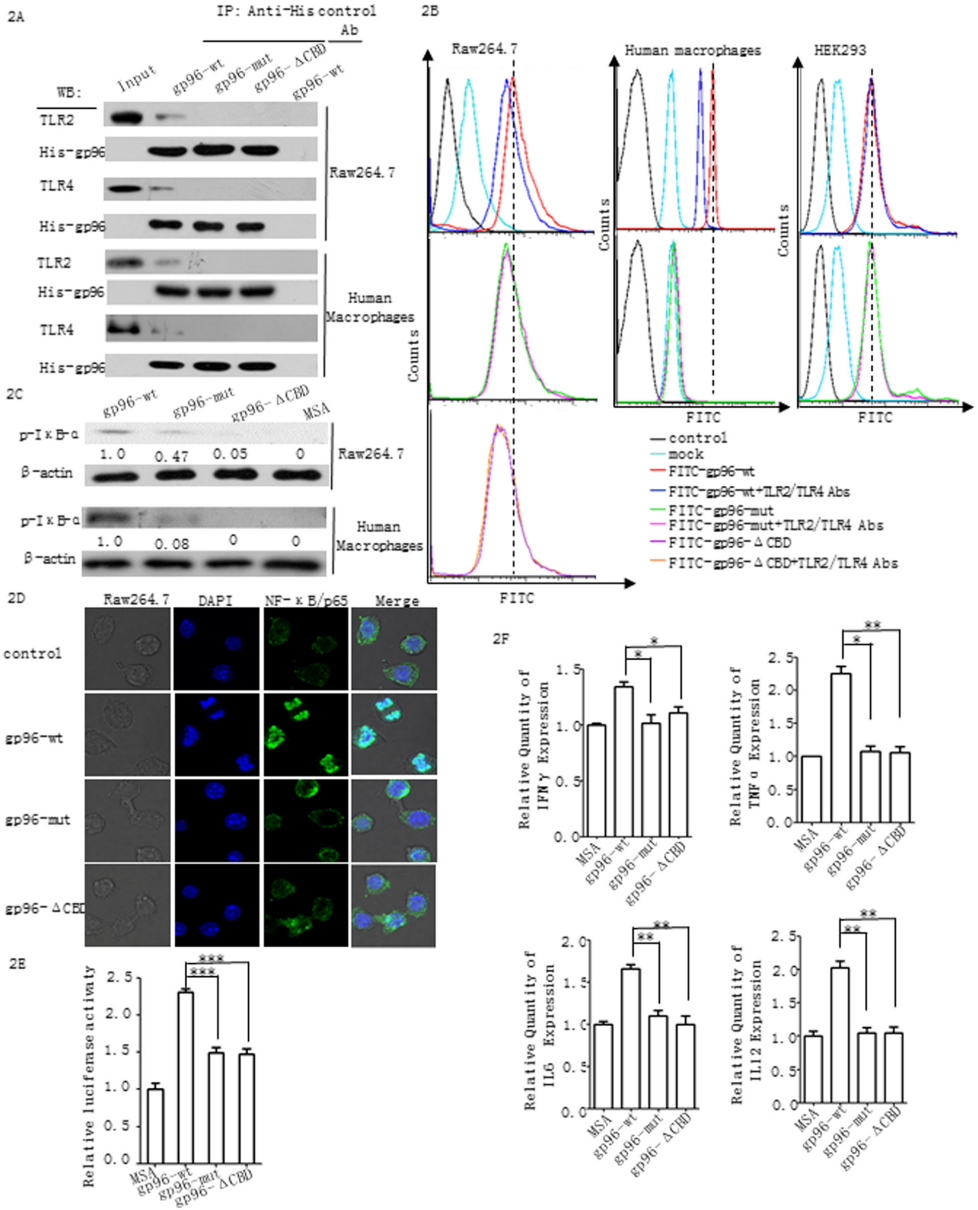
Gp96 mutants in the client-binding domain lose TLRs activation capacity. (A) Co-immunoprecipitation of His-tagged recombinant gp96-wt, gp96-mut or gp96-ΔCBD with TLR2 and TLR4 in RAW 264.7 macrophage cells or primary human macrophage cells with the anti-His mAb. (B) The binding of FITC-gp96-wt, FITC-gp96-mut, or FITC-gp96-ΔCBD to RAW 264.7 cells, HEK293 cells or human macrophage cells was analyzed by flow cytometry. The cells were incubated with 100 μg/ml of gp96-wt, gp96-mut or gp96-ΔCBD at 4°C for 4 h. Excessive anti-TLR2 or TLR4 Abs were added for blocking. (C) The phosphor-IκB-α levels in RAW 264.7 cells or in human macrophage cells treated with 100 μg/ml of gp96-wt, gp96-mut or gp96-ΔCBD at 37°Cfor 30 min were detected by western blotting. (D) Nuclear translocation of NF-κB (P65) was detected by immunofluorescent staining in RAW 264.7 cells. (E) RAW 264.7 cells were transfected with the NF-κ B promoter luciferase reporter plasmid and pulsed with 100 μg/ml gp96-wt, gp96-mut, gp96-ΔCBD, or murine serum albumin (MSA) as a control. The relative luciferase activities were then determined after the incubation. (F) The relative IFN-γ, TNFα, IL-6, and IL-12 mRNA levels were measured by real-time PCR in RAW 264.7 cells at 12 h after treatment with 100 μg/ml gp96-wt, gp96-mut or gp96-ΔCBD. Actin mRNA levels were used as the internal control. Data are presented as the mean ± SD from three independent experiments. P<0.05 (*) and P<0.01 (**).

As expected, gp96-wt but not gp96-mut or gp96-ΔCBD could be co-immunoprecipitated with endogenous TLR2 and TLR4 (Fig 2A). FACS analysis also revealed much higher binding of wild type gp96 with RAW 264.7 macrophage cells and primary human macrophages than the gp96 mutants (Fig 2B). Importantly, the presence of TLR2 and TLR4 Abs interfered with wild type gp96 binding on cells, whereas they had no effect on the binding of mutated gp96. In addition, similar binding capacity of wild type and mutant gp96 was observed on TLR2/4 null/null human embryonic kidney (HEK) 293 cells [17]. These data validate that the gp96 mutants partially lose the ability to bind to TLR2 and TLR4 on macrophage cells. Accordingly, much weaker activation of the NF-κB signaling pathway was observed in the mutant gp96-stimulated RAW 264.7 cells compared to the wild type gp96-treated cells, as indicated by western blotting of phosphor-IκB-α (Fig 2C), immunofluorescence staining analysis of the nuclear translocation of NF-κB (Fig 2D), and a NF-κB promoter luciferase reporter assay (Fig 2E). The IFN-γ, TNFα, IL-6, and IL-12 gene transcription levels were then measured after gp96 stimulation. As shown in Fig 2F, significantly lower levels of all four mRNAs were observed after treatment with gp96-mut or gp96-ΔCBD compared to gp96-wt.

**Fig 3:**
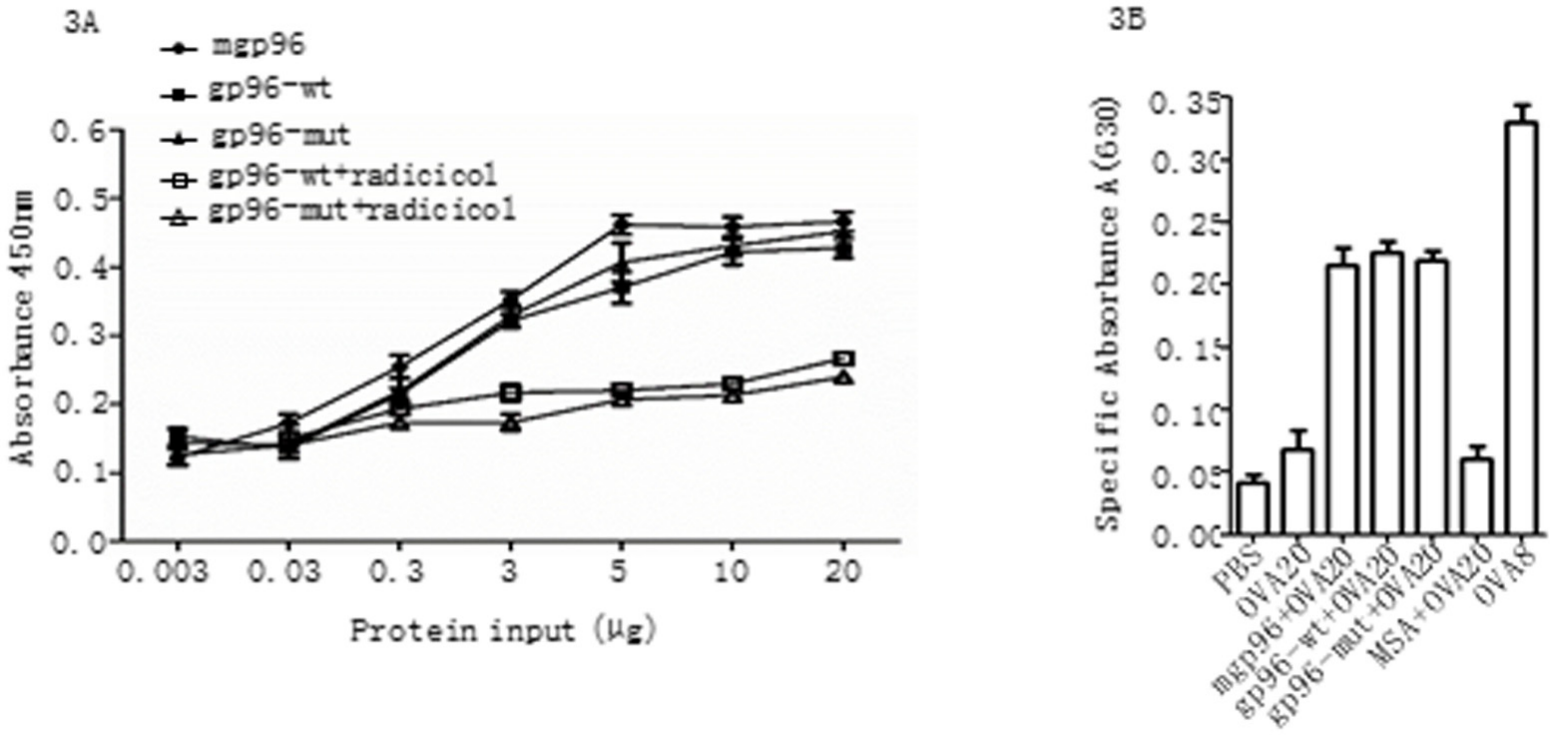
Peptide binding and re-presentation of gp96-chaperoned peptides in vitro are not affect by mutations in the CBC region. (A) Dose-dependent binding of HBcAg_87–95_ peptide to gp96-wt, gp96-mut or native mouse gp96 (mgp96) as a positive control was analyzed in a 96-well plate assay. Inhibition of peptide binding was performed by incubation with 300 μM pan-HSP90 inhibitor radicicol. (B) Cross-presentation of mgp96-, gp96-wt-, or gp96-mut-chaperoned peptides by MHC I of K41 cells, an SV-40 transformed H-2^b^ fibroblast line, was tested by incubation of cells with gp96 complexed to precursor ova20 peptide. The color intensity as an indication of B3Z activation was evaluated using an ELISA reader. Response by B3Z was measured as specific absorbance at 630 nm. Controls include cells with OVA20, MSA complexed OVA20, PBS, or ova 8mer peptide (OVA8). Experiments were performed for three times. Results are presented as mean ± SD.

Meanwhile, the antigen presentation capacity of mutated gp96 was compared in parallel to the wild type protein. The biotinylated peptide HBcAg_87–95_, an HLA-A11-restricted CTL epitope of HBV that naturally binds to gp96 in HBV-infected patients [19], was immobilized in 96-well streptavidin plates. As shown in Fig 3A, both the wild type and mutated gp96 bound to HBcAg_87–95_ in a dose-dependent fashion until a saturation level was reached at a concentration of 50 μg/mL. Of note, the peptide binding activity was largely abrogated by the pan-HSP90 inhibitor radicicol [20]. Next, we examined if the mutated gp96 was functionally competent to cross-present peptide to MHC I molecules using the well-characterized model antigen ovalbumin (OVA), which allows for cross-presentation of the gp96-chaperoned peptides [4, 6, 7]. The wild type or mutated gp96 was mixed with a 20 mer extended peptide (OVA20) that contains the H-2K^b^-restricted epitope SIINFEKL (OVA8). The H-2^b^ fibroblast cell line K41 was first incubated with the gp96-OVA20 complexes and then T cell hybridoma B3Z, which synthesizes β-galactosidase when its T cell receptor engages the OVA8/K^b^ complex. As seen in Fig 3B, similar cross-presentation efficiency of chaperoned OVA20 was observed between gp96-wt and gp96-mut or gp96-ΔCBD by the B3Z T cell stimulation assay. OVA20 peptide alone at a concentration similar to that in the gp96-peptide complexes was poorly cross-presented. These data indicate that the mutated gp96 proteins have equal peptide presentation capacity compared to their wild type counterpart.

### The important role of the TLR binding domain for gp96-induced specific T cell responses

**Fig 4:**
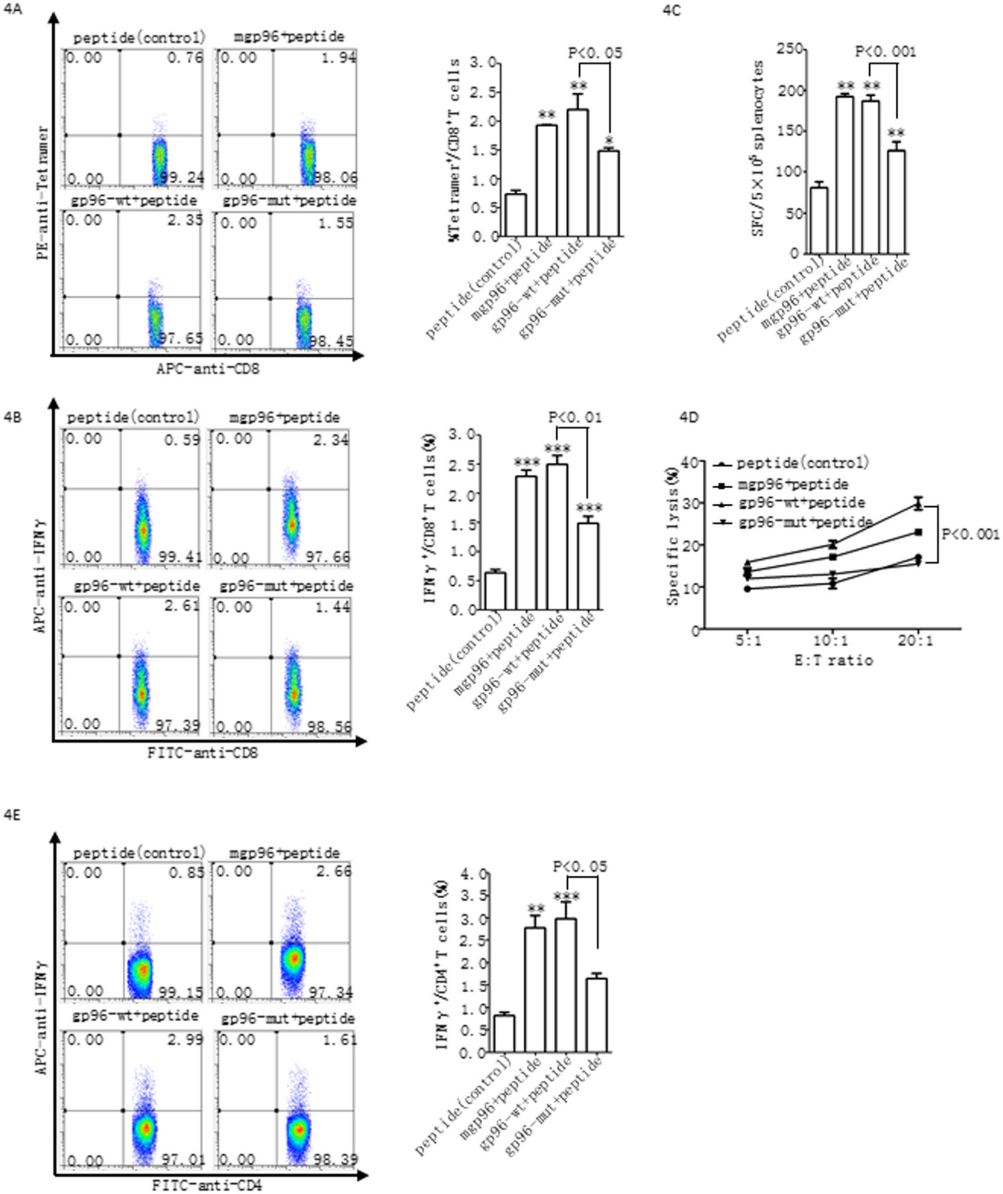
Peptide-specific T-cell response in mice immunized with wild-type or mutant gp96-HBcAg_87–95_ peptide complexes. BALB/c mice were immunized with mgp96, gp96-wt or gp96-mut complexed with HBc_87-95_ peptides three times at weeks 1, 2, and 4, respectively, or with the peptide alone as a control. Each group contains at least five mice. Splenocytes (5×10^5^ cells/well) isolated from mice at week 5 were stimulated with 10 μg/ml HBc_87-95_ peptide. FACS analysis was performed to quantify Tetramer^+^ CD8^+^ (A), IFN-γ^+^ CD8^+^ (B), and IFN-γ^+^CD4^+^ (E) T cell populations. Peptide-specific CTLs were detected by IFN-γ ELISPOT assays (C). The peptide-stimulated splenocytes were incubated with P815 target cells labeled with CFSE and pulsed with HBc_87-95_ peptide. PI staining was then used to measure the killing by FACS analysis (D).*P<0.05 and **P<0.01 compared to control. The data show the mean ± SD of five mice. The data are representative of two independent experiments with similar results.

Next, we comparatively analyzed T cell induction by wild type and mutant gp96. HBc_87–95_ peptide was used to immunize female BALB/c mice with gp96-wt or gp96-mut as an adjuvant for three times at weeks 1, 2, and 4, respectively. Splenocytes were then isolated from mice at week 5 for antigen-specific T cell analysis. Compared to wild type gp96-immunized mice, a significantly lower number of peptide-specific CTL (gp96-mut *vs*. gp96-wt, 1.48±0.11 *vs*. 2.21±0.7, P<0.05) or IFN-γ-secreting CD8^+^ T cells (gp96-mut *vs*. gp96-wt, 1.49±0.42 *vs*. 2.50±0.40, P<0.05) were observed in mutant gp96-immunized mice (Fig 4A and 4B). In addition, compared to the control, immunization with wild type gp96 increased peptide-specific CTL and IFN-secreting CD8^+^ T cells by 3- and 4-fold, respectively, which was much higher than the increase achieved using mutant gp96. Similar results were obtained from the analysis of ELISPOT assays (Fig 4C), the cytotoxicity of CTLs (Fig 4D), and IFN-γ-secreting CD4^+^ T cells (Fig 4E). These results indicate that the interaction with and activation of TLRs by gp96 contributes to gp96-induced antigen-specific T cell responses.

### Impaired tumor rejection by mutant gp96 immunization

**Fig 5:**
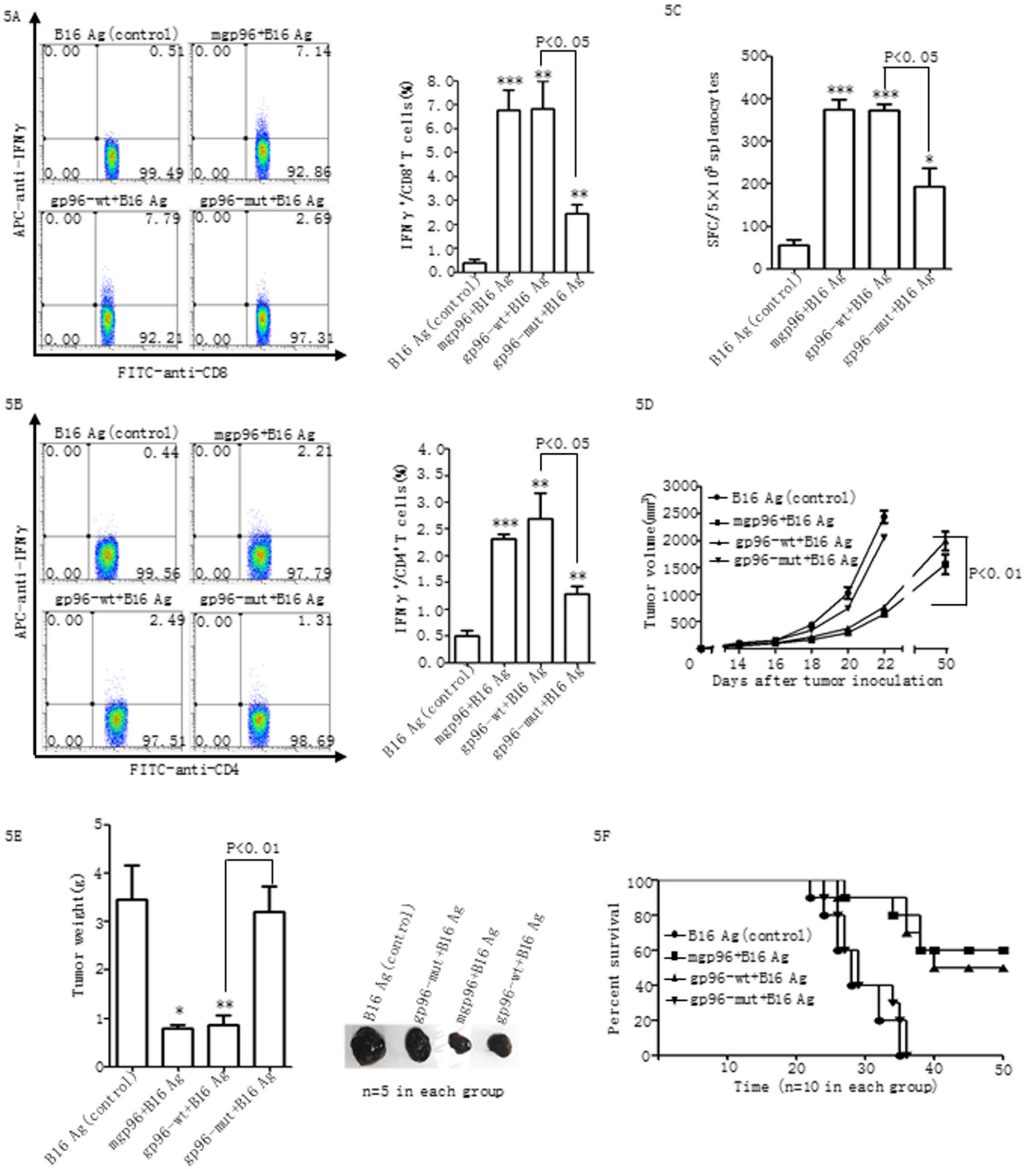
Impaired anti-tumor T cell responses by mutant gp96 immunization. Female C57BL/6 mice immunized three times with mgp96, gp96-wt or gp96-mut complexed with B16 Ag three times at weeks 1, 2, and 4, respectively, or with B16 Ag alone as a control. One week after the third immunization, mice were subcutaneously challenged with 5×10^4^ B16-F10 cells. Each group contains 15 mice. Intracellular cytokine staining for IFN-γ, to quantify the IFN-γ^+^CD8^+^ (A) and IFN-γ^+^CD4^+^ (B) T cell populations of splenoctyes from immunized mice was performed using FACS analysis. Splenocytes (5×10^5^ cells/well) from immunized mice were stimulated with B16-F10 whole cell lysates antigens or BSA for background evaluation and assayed by IFN-γ ELISPOT assays (C). Tumor diameter was measured at two-day intervals (D). Tumor weight was measured when mice were sacrificed on day 22 after tumor injection, and representative images of subcutaneous tumors from B16 Ag, or mgp96, or gp96-wt, or gp96-mut vaccine-treated mice are shown in the right panel (E). Mouse survival was calculated using the Kaplan-Meier plot (F). The data show the mean ± SD of five mice. *P<0.05 and **P<0.01 compared to control. The data are representative of two independent experiments with similar results.

As the CTL response plays a major role in tumor immunity elicited by gp96–tumor Ag complexes, we examined the effect of immunization with wild type or mutant gp96 on tumor rejection as a direct *in vivo* read-out of the CTL activity induced by gp96. We found that C57BL/6 mice immunized with the gp96-mut-B16 Ag complexes displayed a significant decrease in IFN-γ-producing CD8^+^ T cells (approximately 3-fold) (Fig 5A), IFN-γ-producing CD4^+^ T cells (approximately 2-fold) (Fig 5B), and SFCs in IFN-γ ELISPOT assays (approximately 2-fold) (Fig 5C) compared to the mice immunized with gp96-wt-B16Ag complexes (all P<0.05). As expected, compared to wild type gp96, mice immunized with mutant gp96 vaccine exhibited dramatically increased tumor growth (Fig 5D) and tumor burden (Fig 5E) by 70 and 75%, respectively (tumor size (mm^3^) on the 22^nd^ day after B16 injection: wild type gp96 *vs*. mutant gp96 or B16 Ag alone, 769.6±24.44 *vs*. 2053.5±14.06 or 2440.26±113.49) (both P<0.05). In addition, in contrast to wild type gp96, treatment with mutant gp96 vaccine failed to increase the survival rate of tumor-challenged mice (Fig 5F). These results indicate that the antitumor activity of the gp96 vaccine is at least partially dependent on its interaction with TLRs.

## Discussion

A number of studies validated the immune-modulation and adjuvant activities of gp96, which induces both innate and adaptive immunity. However, the efficiency of gp96-based immunotherapy has been modest [1, 24]. The mechanisms of gp96 activity still deserve further understanding, and evidence from different sources has indicated several alternative mechanisms for gp96-induced T cell responses [25, 26]. Here, we investigated the functional relevance of gp96-mediated innate immunity via TLRs with its antitumor and antiviral T cell immunity. By using TLR-binding domain-mutated gp96, which retained peptide cross-presentation capacity, we demonstrated that the selective deficiency in the gp96-TLRs interaction led to significantly reduced activation of specific T cell responses. As we and other studies [20, 23] have shown that gp96 binds peptides within its N-terminal, it is conceivable that mutation of its binding site with TLRs located within its C-terminal does not affect its peptide presentation ability. Meanwhile, mutation of the TLR-binding domain caused a dramatic decrease in gp96-mediated antitumor activity. We thus have succeeded in dissecting the impact of gp96-TLRs interaction in both innate and adaptive T cell immunoresponses.

Numerous studies have shown that gp96 has the unique capability to associate with antigenic peptides generated within tumor or virus-infected cells, with lengths ranging from 5-mers to ≥25-mers, possibly containing various MHC class I- and class II-restricted epitopes [5, 19, 27]. The N-terminal peptide-binding site of gp96 has been further identified in several studies [20, 23, 28]. After immunization, gp96-antigenic peptide complexes are taken up by macrophages and dendritic cells through CD91 or scavenger receptor-A (SRA), followed by activation of specific CD8^+^ and CD4^+^ T cell responses through presentation of the associated peptides to MHC class I and class II molecules [14, 22]. In the meantime, as one of the most abundant chaperones in the ER, gp96 binds a handful of client polypeptides (*e*.*g*., TLRs, integrins, and HER2) and guides their maturation and assembly into large multimeric protein complexes [8, 10, 29, 30]. Notably, exogenous gp96 interacts with TLRs (TLR-2, TLR-4, and TLR-9) or CD91 on APCs, which triggers activation of their downstream NF-κB and p38 MAPK pathways. This leads to maturation of APCs, with cytokine secretion and upregulated expression of co-stimulatory molecules [9, 11]. In this study, we provide direct evidence showing the inextricable role of gp96-mediated activation of TLRs in its major immunological activity of T cell activation. Given the apparent dependence of potent T cell priming on the gp96-TLRs interaction and activation of APCs, it is critical to explore ways to improve these processes and enhance the capacity of gp96 in TLR activation.

In this study, we show that gp96 mutant within its binding site with TLRs has similar peptide presentation ability as its wild type form (see Fig 3B). As gp96-antigen complexes enter dendritic cells and APCs through gp96 receptor CD91- or/and receptor scavenger receptor-mediated mechanism, and the associated antigens are eventually presented by MHC I and II molecules [22, 31, 32], we hypotheses that mutant gp96 still has the capacity to interact with CD91- or receptor scavenger receptor on the surface of dendritic cells and APCs. This deserves further investigation.

Currently, it is difficult to mechanistically distinguish the exact roles of gp96 in peptide cross-presentation and the activation of APCs during CTL activation. The N-terminal fragment containing the peptide-binding domain partially activates the CTL response relative to the full-length gp96, whereas its C-terminal domain containing the TLR-binding motif does not effectively initiate a T cell response [33]. Moreover, antitumor T cell immunity elicited by gp96 is abrogated by blocking re-presentation of gp96-chaperoned peptides [7]. In addition, we previously showed that efficient transduction and internalization of gp96–peptide complexes into APCs determine the outcome of gp96-based immunotherapy [13]. Taken together, we speculate that while Ag cross-presentation by gp96 plays a central role in priming robust T cell responses, activation of APCs through gp96 signaling via TLRs effectively facilitates this process. This deserves further investigation.

In summary, this study emphasizes the functional importance of gp96-mediated TLR activation in eliciting potent T cell responses. Our results may help to elucidate the T cell activation mechanism of gp96 and facilitate a more efficient approach to improve the immune activity of this unique T cell adjuvant.
